# A qualitative exploration of attitudes to walking in the retirement life change

**DOI:** 10.1186/s12889-022-12853-2

**Published:** 2022-03-09

**Authors:** Aidan Searle, Georgia Herbert, Andy Ness, Charlie Foster, Andrea Waylen, Russell Jago

**Affiliations:** 1 National Institute for Health Research Bristol Biomedical Research Centre, University Hospitals Bristol and Weston NHS Foundation Trust and University of Bristol, Bristol, UK; 2 Bristol Dental School, University of Bristol Dental School, Lower Maudlin Street, Bristol, BS1 2LY UK; 3 Centre for Exercise, Nutrition and Health Sciences, School for Policy Studies, University of Bristol, Social Science Complex, 8 Priory Road, Bristol, BS8 1TZ UK

**Keywords:** Walking, Retirement, Intensity, Cadence, Motivation, Habits, Strategies, Qualitative

## Abstract

**Background:**

Walking is a simple activity that could help to reduce the prevalence of chronic diseases in all populations. Furthermore, an inverse dose–response relationship exists between steps taken and risk of premature death and cardiovascular events in middle-aged and older adults. There is a lack of information on how to effectively engage older adults around retirement age in walking. This qualitative study explored attitudes towards walking in older people with regard to habits, intensity, preferences and strategies for increasing walking behaviour.

**Methods:**

In-depth qualitative interviews were conducted with 26 older adults who were either close to retirement or recently retired. An inductive thematic analysis was conducted.

**Results:**

Three themes were identified from the data; 1) Engagement and perceived value of walking; was focused on the meaning of walking for the participant and the attributes they associate with their walking practice. 2) Integration and connectivity of walking; was focused on how participants integrate walking in their daily lives and whether walking can be practiced as a viable means of connectivity. 3) Strategies to increase walking; was focused on what factors motivate participants in their walking practice and what strategies they perceived to be beneficial to increase walking distance and intensity at an individual level.

**Discussion:**

The views of walking in people of retirement age were represented within 3 key themes. The factors contained in these themes that may influence future walking practice are discussed with regard to future strategies to promote walking in the retirement life change.

**Conclusion:**

It may be beneficial to promote qualitative aspects of walking practice and strive for regularity rather than intensity of walking to accrue the social, psychological and intellectual benefits reported by individuals in the retirement life change.

**Supplementary Information:**

The online version contains supplementary material available at 10.1186/s12889-022-12853-2.

## Background

Physical activity is associated with reduced risk of heart disease, type 2 diabetes as well as some forms of cancer [1]. Physical activity is also associated with improved mental well-being across the life course [2]. All adults should undertake 150–300 min of moderate-intensity, or 75–150 min of vigorous-intensity physical activity, or some equivalent combination of moderate-intensity and vigorous-intensity aerobic physical activity, per week [3]. Older adults (> 65 years) are advised to accumulate 150 min of moderate intensity aerobic activity, building up gradually from current levels. It is also suggested that older adults should strive to break up sedentary periods with light activity when physically possible, or at least with standing, as this has distinct health benefits for older people.

A number of studies have shown that large proportions of adults do not meet physical activity guidelines and that levels of physical activity decrease and sedentary time increase with age [4–6]. For example, among older adults (75 + years of age), only 1 in 10 men and 1 in 20 women in the UK meet the physical activity guidelines [7]. Furthermore, there is a marked age-related decline in the proportion of total moderate and vigorous physical activity attributable to exercise and fitness [8]. A systematic review identified five life changes that can contribute to a decline in physical activity; change in employment status; residence; physical status; relationships; and in family structure. All these life changes may be associated with retirement as it is a period when routines that have been established earlier in life may change or stop completely [9]. For example, retirement may mean the active commute to work is no longer necessary but there may now be time to engage in recreational physical activity. Understanding the factors that are both positively and negatively associated with physical activity around retirement is important for developing effective prevention strategies.

Walking is an activity promoted by the National Health Service (NHS) as a key source of regular physical [1]. A systematic review has shown that the independent impact of walking on reducing all-cause mortality was estimated to be 11% (95% CI 4 to 17%), based on 280,000 participants and 2.6 million person-years [10]. Recent evidence suggests that moderate and/or vigorous leisure walking can also increase the mental health and health perceptions of older adults [11]. Furthermore, a new meta-analysis supports an inverse dose–response relationship between steps taken and risk of premature death and cardiovascular events in middle-aged and older adults [12]. Thus, walking is a simple activity that could help to reduce the prevalence of chronic diseases in all populations [13]. To develop strategies to promote walking around retirement there is a need to understand more about why some older adults engage in walking while others do not. This qualitative study explored attitudes towards walking in older people with regard to habits, intensity, preferences and strategies for increasing walking behaviour.

## Methods

### Research ethics

The study was granted ethical approval from the School for Policy Studies Ethics and Research committee at the University of Bristol on 28^th^ July 2020 (REF SPSREC19/20–108) and written informed consent was received for all participants. All methods were carried out in accordance with relevant guidelines and regulations.

### Design and settings

In-depth qualitative interviews were conducted with older adults who were either close to retirement or recently retired. Participants were individuals aged 50–75 who were either due to retire in the next 12 months or who have been retired for 12 months and who were not engaging in the recommended amount of moderate or vigorous physical activity (MVPA). Prior to invitation for interview participants’ current physical activity was assessed with an online survey to assess self-reported daily/weekly MVPA.

Three different methods were employed sequentially to recruit participants: 1) A press release was posted onto the University of Bristol and Bristol Biomedical Research Centre’s webpages and advertised via Twitter; 2) A number of trade union organisations and professional organisations were approached and requested to share recruitment materials (this approach did not yield any participants); 3) Presentations and conversations were held with local professional and community groups such as Active Older People: Promoting Healthy Life Expectancy (APPHLE) Bristol Health Partners Health Integration Team (December 15^th^ 2020) and overtures to Bristol Active Life Project and the Somali Community in Bristol. All participants who expressed an interest in the study were sent a link to an online information sheet and consent form. All responses were followed-up with an email inviting the individual to take part in the walking study and a reminder email was sent 1–2 weeks later.

### Study tool

An initial topic guide was developed in meetings with the study team (AS, RJ and CF) who have considerable experience in public health and physical activity campaigns in the UK. The guide covered the broad areas of current and past walking behaviour, social engagement, walking environment, reasons for walking, value and benefits of walking as well as potential strategies to promote walking including the use of technology. The final interview topic guide is in supplementary info. 1.

### Interview process and data evaluation

Once the consent form had been completed participants were asked to provide basic demographic information such age, gender, occupation, ethnic identity and daily/weekly activity levels, together with their contact details. Participants were then invited to an interview which were conducted by AS on the telephone (due to Covid-19 restrictions). All interviews were digitally audio-recorded and were between 22–57 min long with a mean of 37 min duration. As recompense for their time participants received a £20 gift voucher.

Audio-recordings of each interview were transcribed verbatim by a university approved transcriber and participants’ transcripts were assigned individual codes.

An inductive thematic analysis was conducted [14] by AS and GH (both experienced qualitative researchers). In this process AS became familiarised with the entire data set through reading and re-reading the transcripts. The next step was to open-code a sub-sample of transcripts (*N* = 6) and AS and GH met to discuss their initial coding until consensus was achieved. Data collection and coding were conducted concurrently following an iterative approach. A final coding frame was developed and NVivo Pro (Version 12.3) was used to input transcripts and code data extracts using this framework. Following further interpretation of the coded data by AS and GH these codes were synthesised to form 3 broader themes.

### Participants

A total of 35 online responses were recorded and interviews conducted between October 2020 –April 2021 when England had varying degrees of limitations on movement due to COVID-19 lockdown regulations. Of these 26 (74%) respondents took part in an interview. Seventeen respondents were female and nine were male. All but one participant was white British with the other participant of British /Caribbean heritage. The age of respondents ranged from 53 to 75 years of age (mean, 62 years, SD = 20) (Table 1). Only 11 respondents were still in employment and comprised mostly professionals i.e. Lecturer, Nurse, Social Worker, Researcher. The 15 retired respondents were also mostly ex-professionals. Some respondents reported osteo-arthritic pain, two were registered blind and one had a diagnosis of Parkinson’s Disease.

**Table 1:** Interview participant characteristics (*n*  = 26)

ID	Gender	Age	Occ /status	Ethnic identity
001	**Female**	**69**	**Retired**	**White British**
002	**Female**	**75**	**Retired**	**White British**
003	**Female**	**59**	**Retired**	**White British**
004	**Female**	**58**	**Retired**	**White British**
005	**Female**	**62**	**Retired**	**White British**
006	**Male**	**63**	**Academic**	**White British**
007	**Male**	**57**	**Lecturer**	**White British**
008	**Male**	**59**	**Retired**	**White British**
009	**Female**	**64**	**Social worker**	**White British**
010	**Female**	**56**	**Retired**	**White British**
011	**Male**	**57**	**Retired**	**White British**
012	**Male**	**75**	**Retired**	**White British**
013	**Female**	**53**	**Editor / Teacher**	**White British**
014	**Male**	**60**	**Retired**	**White British**
015	**Male**	**65**	**Retired**	**White British**
016	**Female**	**57**	**Partnership Liaison Officer**	**White British**
017	**Male**	**67**	**Retired**	**White British**
018	**Male**	**68**	**Retired**	**White British**
019	**Female**	**64**	**Retired**	**White British**
020	**Male**	**59**	**Retired**	**White British**
021	**Female**	**66**	**Retired**	**White British**
022	**Female**	**57**	**Senior Registered Nurse**	**Caribbean**
023	**Female**	**64**	**Unpaid Carer**	**White British**
024	**Female**	**57**	**Senior Lecturer**	**White British**
025	**Male**	**56**	**Self-employed**	**White British**
026	**Female**	**61**	**Data Support Officer**	**White British**

## Results

Three themes were identified within the iterative coding and interpretation of the data which were verified through the consensus based coding approach of AS and GH.

These were 1) Engagement and perceived value of walking; 2) Integration and connectivity of walking; and 3) Strategies to increase walking. Each of the three themes and illustrative quotes are presented below. Additional data are presented in tables to represent the range of participant responses within themes 1, 2 and 3 (Supplementary data).

### 1. Engagement and perceived value of walking

This theme focused on the meaning of walking for the participant and the attributes they associate with their walking practice. Included in this theme are expressions of identity, perceptions and expectations of walking, and perceived gender differences.

#### Identity

Most respondents had walked throughout their lives but did not necessarily identify as walkers:

*I think it’s always been part of my life, it’s always been something I’ve enjoyed doing.* (006 Male, 63 years).

Many respondents differentiated themselves from those perceived as ‘walkers’. For example, some used the term ‘ramblers’ and were perceived to have specialist equipment for hiking:

*I don't think I'd describe myself as a walker because, to me, that's someone that puts on walking boots and hikes, but I very much enjoy walking.* (023, female, 64 years).

Some participants had multiple walking / physical activity identities depending on circumstances, preferences and environment: an urban walker and cyclist and a hiker in the country:

*I’m more likely, in the city, to cycle. I’m a hiker, definitely a hiker. I walk… I mean, I walk everywhere that I don’t cycle, let’s put it that way.* (021, female, 66 years).

#### Perception and benefits of walking

Walking was perceived as both a social practice but also an activity whereby an individual could have agency and choice:

*The thing about going walking is that, if you wanted to, I think there’s quite a lot of opportunity to join organised groups and go that way. Against that, psychologically, if you want to go off and walk on your own it’s not too difficult.* (006, male, 63 years).

For some walking was perceived as a fun activity in and of itself:

*It’s not hiking, you’re just doing urban walking. It’s kind of random and it should be fun….. If you don’t enjoy it, don’t do it. If you do, carry on.* (025, male, 56 years).

*It is good for your balance because I've found, obviously I can't do yoga and Pilates now because of lockdown so, you know the way, you might be going up rough terrain or you might be climbing over a stile, that type of thing is good.* (003, female, 59 years).

The perceived benefits of walking went beyond just the physical. It was also seen as being a social endeavour with shared goals:

*I think, sociable, hoping to lose weight, going with people who hopefully are a similar weight to you or whatever. They may be losing weight together, feeling better, having a chat and possibly going for coffee afterwards, you know*….(003, female, 59 years).

*At least you have the opportunity for people to speak to you, whereas if you're inside there's no opportunity for that. And then, I suppose, what else? It's just like, if you're getting out in the open air, if it's a nice day, you're getting a bit of sun, you're getting a bit of wind on your face.* (012, male, 75 years).

Most respondents cited psychological benefits that could be accrued from walking:

*Well, I think it gets your endorphins up. You feel great afterwards. I think it gives you a more positive attitude to things.* (003, female, 59 years).

*If you are feeling down in the dumps or you have got a really, you know, crap case and things are getting on top of you I think just getting out, regardless of what the weather is like, you feel better afterwards, don’t you?* (009, female, 64 years).

In comparison to other activities walking was seen to yield fewer health benefits than other sports:

*I know walking doesn’t have huge, sort of, health benefits in comparison with cycling, or squash, or racquetball, but it’s certainly better than nothing.* (007, male, 59 years).

*I used to find that I had my more creative thoughts when I was just walking because it was more of a meditative thing, in some way*. (001, female, 69 years).

Other respondents linked their walking practice with their professional identity and personal interests:

*I’m a biologist and, actually, my first degree was in botany. There’s nothing I love more, actually, than being outside. I would love to live somewhere much more rural so I was kind of immersed in it. When I go on holiday, I much prefer going to places where there are fewer people and much more nature. So I love being in nature and I love doing the walking* (034, female, 54 years).

#### Gender and walking

Gender was a determinant in walking practice such that women were often driven by opportunities for mental health benefits from experiencing the natural environment:

*It’s got great mental health benefits being out in nature and seeing the countryside and plants and trees and animals.* (005, female, 62 years).

However, this was view was tempered by urban safety concerns suggesting a preference for group walking:

*Well, a big barrier here is you can do a bit of a pavement walk and if you want to walk out more into the country, you do worry about who you might meet, and especially if you're a woman alone. Whereas if you are in a little walking group – not one where you have to wear hiking books, but just a walking group – there is safety numbers*. (023, female, 64 years).

Women also cited that they were socially conditioned and had internalised ideas of the acceptability and social perceptions of lone walking:

*You just get socialised that way. It’s like I would never… Men go and sit in the pub on their own but women don’t sit in the pub on their own because it’s perceived differently. I don’t think about it in those terms but I guess I’ve probably internalised some of that stuff and that’s probably part of the reason.* (024, female, 57 years).

Social norms led some men to feel that walking alone could be perceived negatively by members of the public:

*I think there is this misconception with men, if they are walking around they are up to no good.* (008, male, 59 years).

However, men were not deterred by such perceptions of others as they generally preferred the urban environment to randomly explore and observe human activity and sites of interest:

*I think, for me, part of the walking thing is you see human activity, it’s there around you….It’s the randomness of you don’t have to all be planned your precise route. I think this idea of randomness and just exploring is good for us actually.* (025, male, 56 years).

*With the city you tend to look at buildings, people, shops, and all the other good things, so there is plenty going on.* (008, male, 59 years).

### 2. Integration and connectivity of walking

This theme focused on how participants integrate walking in their daily lives and whether walking can be conceived of or practiced as a viable means of connectivity. This theme also includes perceptions of ‘purposeful’ and ‘incidental’ walking and the implications of ageing.

The purpose of walking given by participants included connectivity and as a means of transportation between two places:

*Main purpose? Just getting out from A to B, avoiding the buses, because I had a bit of turn on one of the buses when I was ill, back in the day, some 10 years ago now. I was not happy getting on a bus.* (020, male, 59 years).

*Because I need to do a walk every day, and sometimes I feel silly walking on my own, I will factor it into doing something. For example, today I did have an appointment, so I walked to the appointment rather than going in the car.* (005, female, 62 years).

However, some would not engage in walking for the sake of a walk:

*No, I would find it difficult going for a walk for the sake of a walk, unless I am going to something specific or I am with somebody else. In the past I always had a dog, so I had to go for walks. I do not have a dog now.* (001, female, 69 years).

Walking was perceived to have the potential to be integrated into daily life more easily than other forms of physical activity such as cycling due to not needing special equipment and the opportunity to see more:

*I think walking is just easier. You haven’t got to put any special clothes on, dry yourself off or anything. You can just go out your front door, go where you are and you can just explore. It’s absolutely wonderful. It does give you a little bit of variety and also you see your place, your urban place, you see it differently because your pace is different. …. with a walk, for me, I’m exploring more.* (025, male, 56 years).

#### Ageing and walking

Respondents spoke of their family and friends who have developed long-term conditions through ageing which was a seen as something which could be thwarted by engaging in physical activity. However, one respondent did not see ageing as an excuse not to engage in activities:

*I suppose some of the people I know, friends and family, they’ve got different types of long-term conditions. That includes, unfortunately, rheumatoid arthritis, musculoskeletal conditions, I don’t… Some of that stuff comes with ageing, which is quite common. I’m fortunate that I don’t have any of those conditions. I want to maintain… I want to be supple, I’m not going to do that by sitting in a chair. As I’ve said, I’m retired so one thing I’ve got is an abundance of time. So I’ve got no excuses not to engage in activities.* (015, male, 65 years).

Conversely, others acknowledged that physical activity could help preserve the ageing body:

*We know the human body ages, we’ll all age. Your brain will age, your eyes will age, your body will age. You’ve got to look after yourself. Part of looking after yourself could be going for walks* (025, male, 56 years).

However, even if walking had been integral in the life of respondents’, issues such as declining stamina had imposed limitations:

*I mean, it’s been a part of my life for a very long time. But I thought I’d be able to push my stamina and increase my distance, but it’s proving hard, because of the pain and because of running out of energy.* (024, female, 57 years).

Some female respondents saw retirement as an opportunity to spend time engaging in physical activity with partners who were already retired:

*Yes, it would be a longer walk. And the other thing, probably in retirement, is joining a walking group. Which I’ve never considered doing. …. We enjoy one another’s company and we don’t get that much time together. And recognising that he is older than me, then I want to maximise as much time as I can have with him.* (026, female, 61 years).

*I mean he still works full-time as a carpenter and he is nearly 69. But he goes out for long walks every weekend. He probably walks more than me. Yes, he will go out regardless. We will go out more together when we both pack in next year. Anyway that’s the plan.* (009, female, 64 years).

### 3. Strategies to increase walking

This theme focused on what factors motivate participants in their walking practice and what strategies they perceived have been or potentially beneficial to increase walking distance and intensity at an individual level.

Motivation for walking was often perceived primarily as a qualitative and cognitive experience that was not contingent on measurement or intensity:

*I don’t want to know what my heart rate is or something like that. What I like to be able to do is to know I’ve walked three, four, hours or whatever, enjoyed myself, and come back in one piece.* (006, male, 63 years).

Participants preparing for a walk required initial physical and cognitive energy for the walk to be realised:

*The initiation energy for me to do a craft project in the house is much lower than the initiation energy for me to, the activation energy for me to, get my shoes on, think about where I’m going to go, work out how I’m going to get there, all of that stuff. So there’re lots of moving parts that I need to consider before I can go out for what I would consider a walk, in inverted commas.* (024, female, 57 years).

Male respondents were more likely to refer to step counting and found it demotivating if they were not able to achieve the recommended 10,000 steps per day. For some it could be counter-productive or lead to a sense of underachievement:

*I suppose I could improve my steps. They do need attention, I must admit. Because how I look at is I’m letting the side down, if I’m not doing my steps, 10,000 steps a day, if I’m only doing 2,500, 3,000. I’m letting the side down. So, I need to definitely improve on that, that’s a given. I need to improve on my step ratio. And because there is so much pressure that you ought to do this, you ought to do that, you ought to do 10,000 steps, but actually it can just make you feel guilty and lower your self-esteem*. (004, female, 58 years).

It was something to aim for, but most people tried to strike a compromise with regard to step counting and aimed for lower:

*I just wanted something to aim for… I think with my pedometer on my phone, it says I’m aiming for an average daily walking rate of 6,000. If I can make that, I’ll be happier.* (020, male, 59 years).

However, one respondent found step counting to be motivational to the extent they were exceeding the recommended steps:

*Yes, basically I started off in 2017 I got a work mobile phone; I got the app down and basically monitored the steps. The first year, 2017, I did about 3,500 a day, and in 2018 I stepped that onto 7,500 a day. In 2019 I got up to 11,000 and then thought it was getting a bit silly, actually.* (008, male, 59 years).

*I don’t know, you might have this thing where the app is saying, “I need to do this many thousand paces a week and I’ve only done this much, I feel terrible.”* (025, male, 56 years).

Other motivational strategies could be based on distraction such as an individuals’ personal interests such as bird watching or listening to podcasts:

*I think sometimes if people are walking and they are interested in, say, birds, then they will find it easier. I suppose people could actually find out how best they like it, whether it is walking in groups or whether they are walking with some other purpose, or whether they are using the time, as I said, to listen to podcasts, getting out of the house, and you do not have to do anything else.* (001, female, 69 years).

There was an awareness of slowing down as the aging process advanced and for respondents to find a comfortable pace in their walking practice. This awareness impacted on the approaches that could be used to promote increased walking at this life stage:

*I don’t go fast or anything, I’m not one of these power walkers or anything. It’s just ambling, I go at the pace I feel comfortable with.* (025, male, 56 years).

Challenging and increasing hill walking did not feature in accounts of urban walking and was confined to settings on a treadmill:

*I mean, if it was a treadmill, rather than it being flat, I'd put it on about level nine, so you could really tell that you were walking uphill. And then I'd try and get a pace which was not running but it was a fairly decent pace, let's put it that way. And I'd try and do that for about… erm, like I said, you'd probably do 20 min like that, but you'd do sort of maybe five minutes warming up and five minutes cooling down. And I was probably doing that a couple of times a week, maybe three at the outside.* (012, male, 75 years).

Listening to podcasts was also a strategy used by some respondents to encourage them to get out for a walk:

*What I was beginning to do, and this actually would encourage me to walk more… This has now encouraged me to perhaps walk, is I have been listening to podcasts. Yes, that is definitely something I would be doing, or maybe I can still do.* (026, female, 61 years).

#### Social prescribing

Health professionals such as GPs were considered to be well placed for the promotion of walking in the context of ‘social prescribing’ schemes. These schemes are promoted by a number of government and third sector agencies. However, much of the data in this regard was in the context of specific cases and / or for mental health and lifestyle conditions rather than public health:

*And I think that's the programme that they've brought in at VitaMinds, which is obviously- people are going on the pathway for CBT and other things, PTSD, whatever. And what they're finding is that people are- if they plug them into that, it's quite gentle, it's quite prescriptive and people are scoring how they feel…* (016, female, 57 years).

*There is a new young GP here and he's full of ideas and all the latest stuff. With all this social prescribing that's coming in with the NHS and GP surgeries, I'm sure that will be good for getting people to not go to things where they sit around and have tea and cake, but maybe some fresh-air things.* (023, female, 64 years).

Some respondents also considered social prescribing as a strategy for preparing for retirement:

*I can see that a lot of people wouldn’t. I think what would be good for people who are thinking about retiring… If they want to walk, if walking is what they want to do, I think it would be good for them to find some sort of group to join because what’s difficult is to meet people, and stuff, once you’re retired. It would combine the two and might motivate them to do both.* (019, female, 64 years).

Other strategies to increase walking included creating walking groups with a purpose such as exploring the archaeology of an area, and a pub stop:

*I would be attracted if there was a slow walkers’ group, for want of a better way of saying it. I also get very interested if there is a purpose to it, so, for instance, the industrial archaeology of the area, or a focal point. And for a longer walk, I would be really interested if there was, say, a pub stop in the middle.* (016, female, 57 years).

In a similar vein it was considered that historical photography could create the impetus for exploratory walks by revisiting the sites of the original photographs to compare with the present context. This could be a strategy facilitated by mobile phone apps and /or maps.

*I like those old black and white photos. That’s interesting, so that’s how it was. Then you can go there with a specific purpose to see a place, you’ve got a photo and you compare this is* how *It is now. It’s like a then and now thing. Which give you a meaning a purpose for your walk, which is actually really interesting. Maybe there could be stuff through mobile phones. I personally would prefer a small map with a nice little route would be quite good.* (025, male, 56 years).

Walking with dogs was seen as a strategy for engagement in walking and was also an activity that could lead to creative and social outcomes:

*If you were just asking me about exercise and a dog, yes, very good because it gives you a sense of purpose, it gives you a commitment, it can lead to social outcomes because you meet other dog walkers, it can be fun if your dog is frolicking around and being silly, that is just fun…* (001, female, 69 years).

## Discussion

This study reports the findings of in-depth qualitative interviews exploring attitudes towards walking in older people about habits, intensity, preferences and strategies for increasing walking behaviour. Although most respondents identified as ‘walkers’ there was an apparent distinction such that serious walkers were ‘ramblers’ or people that wore specialist clothing, and was, therefore, an identity typically ascribed to others. Even if identifying as a ‘walker’ there were sub-groups within this identity such that respondents differentiated themselves from those that appreciate nature versus the urban, built/and or historical environment. A number of the participants who self-identified as ‘walkers’ also identified as ‘cyclists’ or ‘runners’ such that these were attributes that served in creating multiple identities for themselves as ‘active’ people. Furthermore, respondents’ appraisal of walking could be dichotomised as ‘incidental’ or walking for connectivity (e.g., integration of walking with another activity such as shopping) and ‘purposeful’ walking. For example, walking to the village shops would be ‘incidental’ to shopping whereas walking for perceived pleasure or accrual of health benefits would be a ‘purposeful’ event in and of itself. Respondents who framed walking as purposeful were more likely to consider themselves as ‘walkers’ and favour a rural environment. This perception of walking fits with the findings of a review reporting an overlap in the environmental attributes found to be associated with walking for connectivity and attributes associated with walking for exercise or recreation [15]. In addition, our respondents shared mixed views of walking, if they identified as walkers or not, and similar to other recent qualitative findings reported by Cavill et al., [16]. In this study walking was often something that was not included in respondents’ initial definitions of activity, either because they did not think it was not of sufficient intensity, or because they just saw it as a means of transportation. These walking identities may have been more pertinent in respondents’ past, but this activity may have rescinded due to effects of ageing and /or retirement. Respondents also attributed the onset of pain and reduced stamina to the ageing body even if walking had been integral in their past. Similarly, life events had stopped previous activity including walking in respondents interviewed in Cavill et al.’s study [16].

Despite the onset of age some respondents were looking to increase speed and stamina in walking but were not able to envisage how this change could be achieved. In this regard walking was considered to be a low impact activity that led some respondents to re-engage with walking due to a lowered perceived risk of injury and therefore it could provide the base for future, more intense or longer walking as well as other forms of physical activity. It was clear that walking was not perceived in the same way as other physical activity due to the ubiquitous and multiple contexts that walking occupies. Thus, walking was a way of both staying socially involved whilst continuing a ‘work’ ethic into retirement. The perceived value of walking was coherent in that most respondents reported experiencing benefits of walking such as the physiological and psychological benefits that could be accrued in the short and longer term.

### Social norms and walking practice

Social norms were perceived to mitigate walking practice. Women were more motivated by the social aspect of walking whereas men generally preferred lone walking practice. Women generally preferred social walking due to safety concerns and social norms and provided a space to talk about their lives away from significant others. In addition, women respondents stated that they were conditioned to not walk alone and this exacerbated feelings of self-consciousness in addition to safety concerns. This finding is consistent with a systematic review of quantitative data [17] which found that more women than males walk for leisure, but the gender difference appears to reverse as age increases. Taking all ages together, there was no consistent gender difference in walking for transport or in total walking, although the small number of studies reporting on walking to undertake errands suggested that more women than men walk for this purpose.

There were also internal and external tensions that needed to be reconciled in respondents such that individual perceptions of ability could be at odds with the location, topography, season and other personal interests. Assuming that walking is primarily seen as a social practice walking it is not conceptualised as a singular behaviour but as a diverse “everyday practice of social life” [18] and maybe best understood as “an inherently sociable engagement between self and environment” [19] which would then suggest that lone walking is a social practice.

### Strategies to increase walking practice

Many respondents reported that the value of walking was contingent on opportunities for social intercourse and/or engagement with the urban or rural environment. This perception has implications for the promotion of approaches that may strive to build the intensity (speed) or challenge (incline or duration) of walking. The data here suggest that it may be beneficial to promote these qualitative aspects of walking practice and strive for regularity rather than intensity of walking in alignment with these perceived benefits of walking. This is supported by a review of cadence (steps/min) studies showing that mean steps/min represents a daily average value that is shaped by the naturally large amount of time spent at zero cadence (both individually and on a population level; 19). Relative to the approximately 1000 waking minutes available in a day, it is was apparent that for most people, daily continuous, rhythmic walking of at least moderate intensity is quite rare. Cadence distribution was also more likely to approximate normality than conventional time-above-threshold metrics (i.e., time spent at or above 100 steps/min), because everyone has a score above zero. Thus, it may be more meaningful to categorise measures of cadence according to whether or not an individual accumulates any time at > 100 steps/min and practiced in a context that is meaningful to and feasible for individuals. As such, guidance on walking cadence could form part of future strategies to promote walking intensity around retirement age.

From a theoretical perspective quantitative studies report that habit strength is consistently positively correlated with behavioural frequency of an activity [20, 21] and may bridge the ‘gap’ between intention and behaviour. The present qualitative data would suggest that walking behaviour was contingent on personal and social norms which would support both habituation and habit strength approaches to increasing walking. The data presented here are consistent with the COM-B model of behaviour change [22] as it would suggest a focus on individuals’ perceived capability, opportunity and motivation for walking. Providing an opportunity that encapsulates both physical access and social acceptability. The COM-B model could also be integrated with socio-ecological approaches which position individual cognitions as the mediator of external factors such as the built environment. A recent review of socioecological models in explaining physical activity (PA) has highlighted that a connected and accessible environment for PA may be important for an individuals’ self-volition. In addition, the public health implications of individual cognitions and how habits connect with the built environment may serve to explain engagement in PA. Therefore, investments in infrastructure, particularly those that improve connectivity and accessibility, may be critical to successful promotion of health behaviours such as walking at the individual-level [23].

It is also suggested that others’ behaviour can influence an individual’s behaviour as a cue to action and enforce patterns of social control, or place constraints on individual choice through interpersonal relationships. These factors are therefore key considerations when designing a walking program around the retirement life change.

Social Prescribing initiatives have been widely implemented in the UK National Health Service in primary care [24]. Social prescribing links patients with non-medical needs to sources of support provided by the community and voluntary sector to help improve their health and wellbeing [24]. The present qualitative data revealed that interventions may be most effective if tailored to individual preferences; for example walking groups for those favouring group activities and opportunities to talk while they walk; provision of maps and safe routes for people who want to walk alone or to incorporate walking into their daily routines as a means of connectivity. If individual approaches to promoting walking, i.e., Social Prescribing, are to be successful this study suggests that individual choice of activity would be favoured over prescription.

### Implications for the promotion of walking

Table 2 highlights the key findings from this study and the implications for promoting walking around retirement age. A central message is the promotion of the psychological benefits and peer support for walking above any physical benefits. In addition, conditioning, gender norms, ageing and connectivity were perceived to mitigate respondents walking practice and need to be considered when designing appealing walking programs for older adults. With regard to increasing walking practice the qualitative and cognitive experience was seen as more important than measurement such as steps/pace. The data here suggest that it may be beneficial to promote these qualitative aspects of walking practice and strive for regularity rather than intensity of walking in alignment with these perceived benefits of walking. As such building habit and routine and then maintaining the walking routine both in leisure and lifestyle activities would appear to be essential for the promotion of walking at the retirement life change.

**Table 2:**
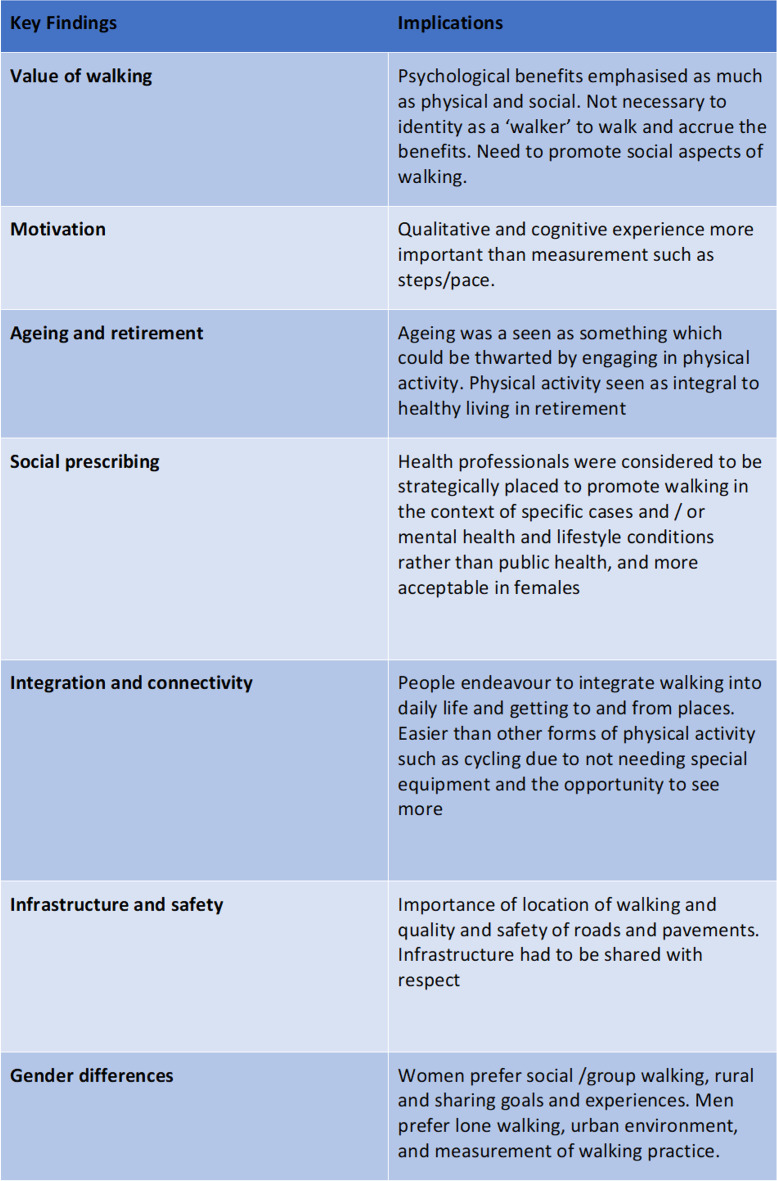
Key findings and implications

### Limitations

The qualitative interviews followed an online survey that was distributed through university press and social media platforms, community organisations and allied health partners which limited the generalisability of the sample. Interviews were conducted in a period when the UK was in various different stages of national and local COVID-19 lockdowns which may have influenced the ability of respondents to walk despite alluding to be active people. Responses to the survey were mainly female, (ex) professional, urban Bristol dwellers despite the online / national coverage. As the research was designed prior to COVID-19 restrictions and subsequently the impact of the government guidance led to revisions been made to the topic guide and may have temporarily influenced individuals’ walking behaviour and narratives. It should also be noted that the wide age-range of participants has implications for the ability to engage in walking due to age induced morbidity and perceived ability to engage in walking. There were also difficulties in recruiting participants to the study, particularly with regard to representation of ethnic minority groups and those of lower socio-economic status thus limiting the generalizability of the findings. With regard to reflexivity it is acknowledged that the lead author, interviewer and primary data analyst was an active walker at the time of data collection. Although this was not disclosed to participants it may have influenced the interview process and interpretation of data.

Implications for further studies include the need to conduct qualitative studies in lower socioeconomic groups and /or rural communities. Prospective studies pre- and post- retirement would serve in delineating how significant life changes such as retirement shape attitudes to and engagement in walking, ideally not in a pandemic induced lockdown.

## Conclusion

The views of walking in people of retirement age are represented within 3 key themes and the limitations factors contained in these views impose on present and future walking practice. It may be beneficial to promote qualitative aspects of walking practice and strive for regularity rather than intensity of walking to accrue the social, psychological and intellectual benefits reported by individuals in the retirement life change.

## Supplementary Information

**Additional file1.** Topic guide.

**Additional file2. **

## Data Availability

The datasets used and/or analysed during the current study are available from the corresponding author on reasonable request.

## Abbreviation

NHS: National Health Service
