# Supplementary material for: A qualitative exploration of attitudes to walking in the retirement life change
Source: BMC Public Health. 2022 Mar 9;22:472. doi: 10.1186/s12889-022-12853-2 (PMC8905568; doi:10.1186/s12889-022-12853-2)
Supplement: Supplementary file 1 — Additional file1. Topic guide. [file 12889_2022_12853_MOESM1_ESM.docx]

| **Supplementary info – Topic guide** |
| --- |
|  |
| **Introduction / background** |
| Are you employed or in retirement? |
| Do you live alone or with others? |
| Do you have any interests or hobbies? |
| Do you have any long-term health conditions? |
| How did you find out about this study? |
|  |
| **Current walking behaviour** |
| Would you describe yourself as a walker? |
| Can you describe your current walking behaviour? |
| How long is a typical walk (mins/hours/distance)? |
| How long have you been walking – has this increased since Covid-19? |
| What is the purpose of walk – weight management, social, coincidental /shopping? |
| Do you wear any special clothing/equipment to walk – shoes, sticks, rainwear etc? |
| Can you give an example of a recent walk? |
| What are your reasons for walking; |
| Prompt: active travel, leisure, relaxation |
| Do you walk alone or with others? |
| What are your thoughts about walking; |
| - Alone? |
| With others? Preference for single sexed or mixed groups? |
|  |
| Do you walk with dogs? |
| Prompt: If so what are the benefits of walking with dogs? |
| To what extent do you value walking - what benefits or attributes does walking have? |
| Prompt: health benefits, mental, physical, weight, self-image |
| Perceived impact of walking on mental health / wellbeing |
| What are the mechanisms of action for these perceived benefits? |
| To what extent does having conditions that affect your health impact walking? |
| Do you use walking as a means of managing health conditions? |
| What is your preference of walking environment – urban / rural? |
| To what extent does the impact of / interaction with environment affect your walking behaviour? |
| What do think about while walking – personality type / introspective? |
| Potential for increasing walking behaviour |
| What are the barriers to walking or starting to walk? |
| Prompt: physical and psychological |
| What is your view on individual differences in walking duration, speed, etc? |
| What journeys do you currently make and are there possibilities to switch to walking for parts of journeys? |
| Do you use technology such as devices to monitor walking (pace, steps, distance, GPS etc)? |
| Role of retirement on walking / routine – what planning could be done pre-retirement? |
| Do you have any motivational strategies for initiating a walk? |
|  |
| There are approaches to encouraging walking such as ‘walk and talk’ whereby older isolated people are encouraged to walk with a peer – do you have any views on this approach? |
|  |
| **COVID-19 Awareness** |
| In what way has Covid-19 and physical distancing guidance affected your walking behaviour? |
| Would you say you are walking more or less since the COVID-19 lockdown? |
| Do you / would you wear a face mask while walking? |
| If so, in what way does wearing a mask affect your walking behaviour? |
| How do feel about physical distancing while out walking? |
| Finally, are there any issues regarding walking that we have not covered and would like to raise? |
|  |
|  |
