# Supplementary material for: A qualitative exploration of attitudes to walking in the retirement life change
Source: BMC Public Health. 2022 Mar 9;22:472. doi: 10.1186/s12889-022-12853-2 (PMC8905568; doi:10.1186/s12889-022-12853-2)
Supplement: Supplementary file 2 — Additional file2. [file 12889_2022_12853_MOESM2_ESM.docx]

| **Theme 1: supplementary data** | **Engagement and perceived value of walking** | **Participant** |
| --- | --- | --- |
| *Identity* | *I think it’s always been part of my life, it’s always been something I’ve enjoyed doing.* | **006, Male, 63 years** |
|  | *I would only walk to walk. I don’t walk places. You know, I get in the car and… My husband jokes that I wouldn’t park further away than four cars from the supermarket.* | **019, Female, 64 years** |
|  | *I would describe myself as someone who loves walking. Walking I wouldn't be a runner; I would be a walker. So I do enjoy walking.* | **023, Female, 64 years** |
|  | *I can do it and, yes, given the opportunity, I love it. I guess, historically, I would go out for walks with my father but, of course, that’s gone by the by.* | **023, female, 64 years** |
|  |  |  |
|  | *I certainly see myself as having been a regular walker. More recently, it has been a bit of a problem. I really love walking if I’m meeting friends and family.* | **026, female, 61 years** |
| *Perceptions and benefits of walking* | *Well, if it wasn’t enjoyable, we wouldn’t do it, I don’t think* | **024, female, 57 years** |
|  | *Just to try and get a bit fitter, and just because of the place we live. It is a lovely place, and just to get out and enjoy the countryside really.* | **024, female, 57 years** |
|  | *And of course, you're not being isolated either, you're talking out there, you're talking about what’s going on in the world, you're learning all the time. It’s good for your wellbeing, both physical and mental.* | **020, male, 59 years** |
|  | *So, yes, it is a psychological benefit, but it’s not something I tend to think about.* | **007, male, 57 years** |
|  | *Well, I think it gets your endorphins up. You feel great afterwards.* | **003, female, 59 years** |
|  | *I’m not quite sure how to describe that feeling but you feel refreshed, if you like, once you’ve gone out for a walk and back.*  *I need to get out and get about so I can get some fresh air out there. That’s number one, for a start. There’s a weight loss problem I’ve got to get over, that’s two, so that’s the physical side. And of course, the mental wellbeing as well.* | **022, female, 57 years**  **020, female, 64 years** |
|  | *I probably do feel better for having gone out, and it was a different experience, just that I have made the effort.* | **005, female, 62 years** |
|  | *and with nature, I guess. And yes, the fitness side to it. And it’s good for muscles.* | **026, female, 61 years** |
|  | *I like, walking, it doesn't cost anything and that's the thing about it. Going out for a long walk is really healthy.* | **014, male, 60 years** |
|  | *I mean, any form of exercise is good. I can’t think of any form of exercise that’s bad. And walking certainly is the simplest form of exercise.* | **018, male, 67 years** |
|  | *I mean it helps in maintaining physical fitness. It certainly can help towards weight control.* | **006, male, 63 years** |
| *Gender and walking* | *I think a lot of people, especially women, I think, would probably like to join a group. I think group things would help women, like you know the way they go to a leisure centre to a class?* | **003, female, 59 years** |
|  | *A big barrier here is you can do a bit of a pavement walk and if you want to walk out more into the country, you do worry about who you might meet, and especially if you're a woman alone.* | **023, female, 64 years** |
|  | *Well, people will talk about their problems, you know the way? Sometimes relationship problems, or if whether they are going to retire or not, or if their husband is going to retire or not, you know?* | **003, female, 59 years** |
|  | *I hate walking. Walking for walking’s sake, I do not do it if I can avoid it.* | **002, Female, 75 years** |
|  | *I would fully emphasise that I actually like walking by myself. I really do. just to let the thoughts reshuffle in my mind, not to go through tedious conversations and that sort of thing.* | **006, Male, 63 years** |
|  | *If I’m spending social time with somebody while I’m walking then I think that feels like… That feels more like an outcome.* | **024, female, 57 years** |
|  | *For some people I think there’ll be the social angle, For others, I think they might just want to do it themselves. I’m probably a bit of both inclining more towards the solo thing.* | **025, Male, 56 years** |
|  | *It’s no fun doing a long walk on your own. It’s nicer if you’re walking with… Walking with others, I think, is much easier* | **022, female, 57 years** |
|  | *I often walk once a week on my own, at the moment. Occasionally with one or two other people. But yes, normally I’m on my own.* | **017, male, 67 years** |

| **Theme 2: supplementary data** | **Integration and connectivity of walking** | **Participant** |
| --- | --- | --- |
|  | *I choose to walk just to get some fresh air. If I find myself with an errand to run and it’s not too far, where I need to drive, then I’ll opt to walk for the fresh ai* | **022, female 57 years** |
|  | *I factor in some excuse and then I will, as I say, park at the gates and walk or, in the supermarket, won’t park too near the door and walk all around every aisle and…* | **007, male, 57 years** |
|  | *I would walk for the sake of walking, that was the only reason I would walk. I wouldn’t walk to get somewhere or whatever, I would only walk because I was going for a walk.* | **023, female, 64 years** |
|  | *I walk from the car to my office, I walk from the office to the classroom, and then I walk back to the car, and then I walk form the car into the front door of the house. So, there’s so little exercise.* | **003, female 59, retired** |
|  | *Walking is my main mode of transport. I do cycle, but it's really walking that I prefer to do.* | **019, female, 64 years** |
|  | *It’s a great way to see a city, because you can cover a lot more of the ground, but for that reason I think that we will never just entirely cycle, it will always be a mixture of walking and cycling.* | **012, male, 75 years** |
|  | *I would walk for the sake of walking, that was the only reason I would walk. I wouldn’t walk to get somewhere or whatever, I would only walk because I was going for a walk.* | **026, female, 61 years** |
|  | *You're doing it, really, either because you have somewhere to go, or you're doing because you feel you should do it for exercise or just to get out. But in all honesty, you're not relaxed when you're doing it.* | **016, female, 57 years** |
|  | *I got into the habit last year, in the early parts of lockdown, for going for a morning newspaper. Which was great, just to walk into the village and back.* | **001, male, 57 years** |
|  | *Actually, you know, at university too. Just going into work, getting up, walking around the campus, you know, there’s incidental exercise-* | **024, female, 57 years** |
|  | *I would find it difficult going for a walk for the sake of a walk, unless I am going to something specific or I am with somebody else. In the past I always had a dog, so I had to go for walks. I do not have a dog now.* | **025, male, 56 years** |
|  | *I am a Londoner but it’s in an area of London that I didn’t know before. You know, I’ve spent months pushing him [grandchild] to sleep and really relishing in exploring. So I’ll go down different roads and different alleyways and… I’ve just absolutely loved that.* | **021, female, 66 years** |
|  | *I think all the public authorities and others could do to facilitate things and give people opportunities, encourage people and nudge people. If you’re older and you’ve got time on your hands, your physical health is going to be one of those things you’re going to need to think about.* | **018, male, 68 years** |
| *Ageing and walking* | *You know, I try to do as much as I can. I’m aware I’m getting older, so it’s also another thing where I need to keep going.* | **016, female, 57 years** |
|  | *I think it is important for older people, if they can, to keep moving and going out.*  *Your brain will age, your eyes will age, your body will age. You’ve got to look after yourself. Part of looking after yourself could be going for walks* | **002, female, 75 years**  **025, Male, 56 years** |
|  | *I try to do as much as I can. I’m aware I’m getting older, so it’s also another thing where I need to keep going.*  *I thought I’d be able to push my stamina and increase my distance, but it’s proving hard, because of the pain and because of running out of energy.*  *Some days are better than others. Most of the time I can just walk, but it takes a while to get going. And I've got pain in my joints…* | **022, female 57 years**  **004, female 58 years**  **016, female, 57 years** |
|  |  |  |

|  | **Strategies to increase walking** | **Participant** |
| --- | --- | --- |
| **Theme 3: supplementary data** |  |  |
|  | *I suppose people could actually find out how best they like it, whether it is walking in groups or whether they are walking with some other purpose, or whether they are using the time, as I said, to listen to podcasts, getting out of the house, and you do not have to do anything else.* | **001, female, 69 years** |
|  | *If I’ve got something to aim for… I think with my pedometer on my phone, it says I’m aiming for an average daily walking rate of 6,000. If I can make that, I’ll be happier.* | **020, male, 59 years** |
|  | *I don’t know, all these apps and this and that, it doesn’t really do it for me and, you know, all this pressure to do 10,000 steps a day, if you can do it, you can do it but you can't, you can't.* | **003, male, 59 years** |
|  | *No, I’m not going to get a Fitbit or anything like that. Yes, I know we should be doing 10,000 steps a day, but that doesn’t motivate me.* | **026, female, 61 years** |
|  | *I’ve taken some of the stress out of it by not doing that, because I don’t think, right now, step goals are a particular motivation for me.* | **004, female, 58 years** |
|  | *I’m just interested to be part of something different really, to get me to think along, from my point of view, set myself some new targets and challenges* | **009, female, 64 years** |
|  | *I think the way, perhaps, to encourage walking is to actually just illustrate to people the great advantages of it, which are low* | **006, male, 63 years** |
|  | *Low cost, outdoors, brilliant views, and it can be that you can control and organise just about all of it yourself.* | **019, female, 64 years** |
| *Social prescribing* | *Doctors have recommended doing more exercise, not walking specifically.*  *I’m really into social prescribing. I set up the first social prescribing project in the west of England actually.* | **007, male, 57 years**  **021, female, 66 years** |
|  | *I think all the public authorities and others could do to facilitate things and give people opportunities, encourage people and nudge people.*  *For GPs in that area to be able to give patients blue prescriptions to join walking groups that undertake that… You know, walk near to the sea- Because, for some people, it’s very relaxing.* | **025, Male, 56 years**  **015, male, 65 years** |
|  |  |  |
